# Genome Sequence and Antimicrobial Production of *Pseudomonas crudilactis*

**DOI:** 10.17912/micropub.biology.002227

**Published:** 2026-08-12

**Authors:** Madison McMurphy, Arlo Clarke, Hannah Ralls, Stephanie L Mathews

**Affiliations:** 1 Department of Biological Sciences, North Carolina State University, Raleigh, NC, United States; 2 Department of Plant and Microbial Biology, North Carolina State University, Raleigh, NC, United States

## Abstract

*Pseudomonas crudilactis* was isolated from soil collected in Lake Johnson State Park in Raleigh, NC, and screened for antibacterial activity. This isolate inhibited the growth of *Pseudomonas putida* and *Bacillus subtilis* . After isolation and repeated antibacterial activity testing, the isolate was characterized through gram staining as a gram-negative bacillus. Genomic DNA was extracted and sequenced. After assembly and annotation, the genome was analyzed for secondary metabolites production to find 14 genomic regions of antibacterial activity and 5 unique mechanisms: non-ribosomal peptide synthesis (NRPS), ribosomally synthesized and posttranslationally modified peptides (RiPPs), non-ribosomal peptide metallophores, terpene-precursor, and beta lactone.

**Figure 1. Characteristics of MMA91 ( Pseudomonas crudilactis) an antibiotic-producing bacterium isolated from soil:** A. T-streak of MMA91 on LBA after 48 hours of growth at 30℃. B. Antimicrobial clearing of *Pseudomonas putida by* isolate MMA91 on LBA after 48 hours of growth at 30℃. *Lysobacter antibioticus* was also plated as a comparison. The arrow indicates clearing by MMA91. C. Circos genomic map of isolate MMA91 from genomic sequencing. D. Phylogenetic tree depicts isolate MMA91 grouping closely with *Pseudomonas fluorescens* .

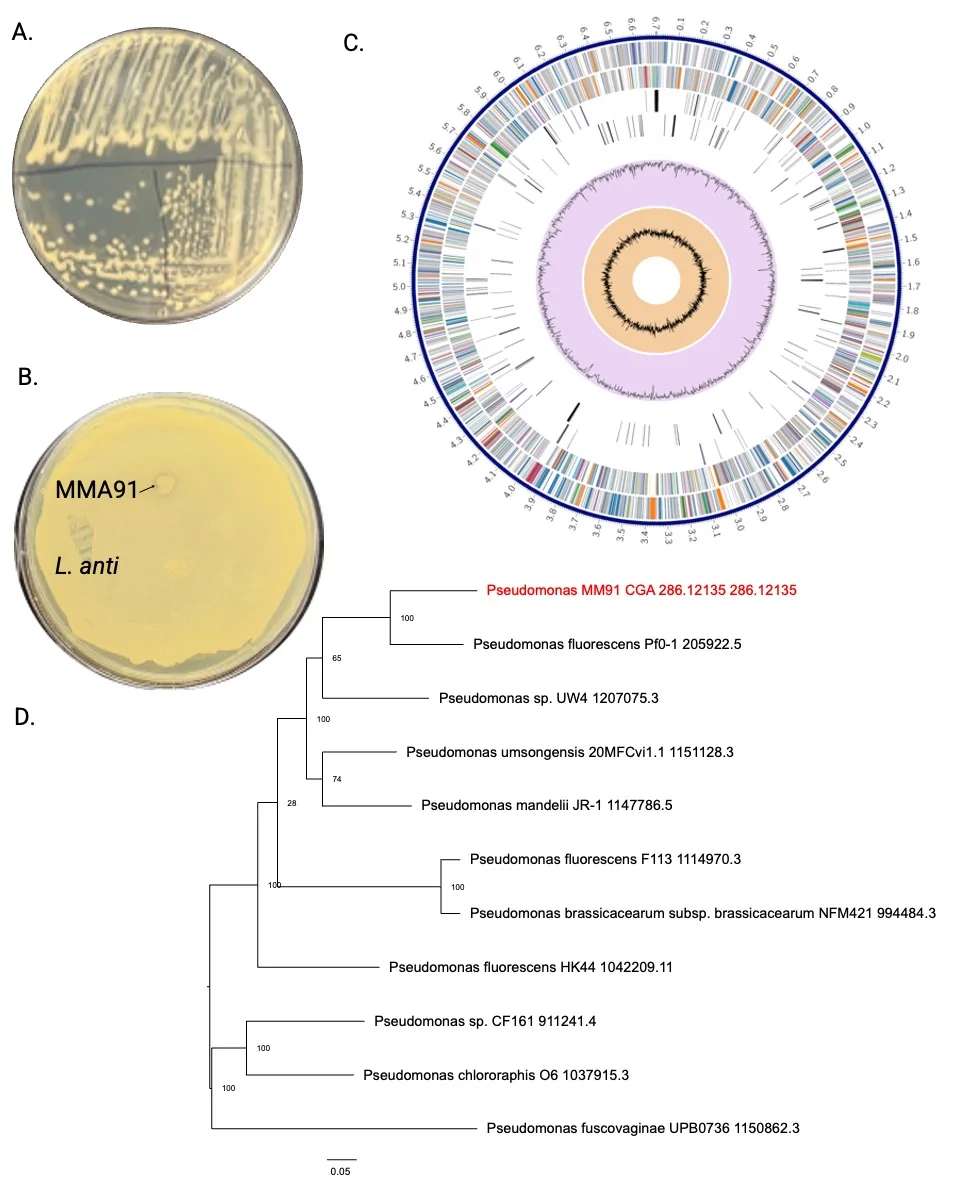



## Description

Antimicrobial resistance (AMR) is a global issue; many bacterial pathogens have become resistant to antibiotics (Wahnou et al., 2026). Effective antibiotics are in a small supply (Ahmed et al., 2024). Researchers have been testing soil microbes in hopes of discovering new and innovative antibiotics to combat antibacterial resistance against these pathogens (Chandra et al., 2017). Soil is a diverse microbial habitat containing bacteria and other microbes that compete for limited nutrients and space. To survive in this environment, many microorganisms have the ability to produce antibiotics to inhibit the growth of neighboring organisms. These naturally occurring antibiotic-producing microorganisms have been the source of many clinically important drugs. Future antibiotic discovery may rely on these same methods but crowd-source through the work of undergraduate students across the world (Miller et al., 2025). 

A soil sample was collected from Raleigh, North Carolina (35.76241, -78,712824), serially diluted, and plated onto LB agar. After incubation for 38 hours at 25℃, individual colonies were selected for further analysis. Soil isolates were screened for activity against safe ESKAPE pathogens utilizing the spread-patch method on LB agar and incubated for 48 hours at 30℃. Inhibition was observed against *Pseudomonas putida* and *Bacillus subtilis* for one isolate: MMA91 ( Figure 1B ). This bacterium was isolated with three successive rounds of t-streaking resulting in round, flat, white colonies with an entire margin ( Figure 1A ). Inhibition of *P. putida* and *B. subtilis* was confirmed after isolation. A Gram stain resulted in pink rods indicative of gram-negative bacteria.

DNA was extracted using DNeasy UltraClean microbial kit (Qiagen) from a liquid culture of MMA91 grown in Luria Broth for 48 hours at 30℃ with shaking at 200 rpm. The DNA was sequencing using Nanopore Native Library Kit and Nanopore MinION. This assembled genome had 1 contig, with the total length of 6,700,725 bp and an average G+C content of 59.13%. Annotation by BV-BRC identified 6,039 protein coding sequences: 1,383 hypothetical proteins and 4,656 proteins with functional assignments. Taxonomy indicates this isolate shares a clade with *Pseudomonas fluorescens* ( Figure 1D ).

Using the genomic sequence, additional analysis was performed using Anti-SMASH to identify 14 secondary metabolite production regions, of which 5 were unique with known antimicrobial properties ( Figure 1C ). Their antimicrobial mechanisms include non-ribosomal peptide synthetase, RiPPs, non-ribosomal peptide metallophores, terpene-precursor, and beta lactone. Non-ribosomal peptide synthetases have the ability to self-assemble, which allows them to adhere better to bacteria and penetrate cells more effectively (Prazdnova et al. 2026). RiPPs are pore-forming peptides that target bacterial cell envelopes (Lia Cao et al. 2021). MMA91 also produced ranthipeptides which are a subset of RiPPs that kill gram-positive bacteria by sequestration of lipid II, pore formation, and binding of phosphatidylethanolamine (Precord et al. 2019). Non-ribosomal peptide metallophores are a secondary metabolite produced by NRPS that bind to iron and starve other microbes of essential iron needed for survival (Prazdnova et al. 2026). Some terpene-precursors have been shown to alter microbial cellular respiration and cause uncoupling of oxidative phosphorylation in the microbes (Huang et al. 2022). They have also been found to interact with the lipophilic tails of intermembrane lipids, altering the transmembrane pathways and affecting the lipid membrane activity (Huang et al. 2022). Beta Lactone is a four-membered ring molecule that inhibits bacterial growth through inactivation of essential enzymes, this includes the inhibition of ClpP protease (Lawrence P. Wackett. 2016). It mimics the action of beta-lactam antibiotics, targeting serine-dependent enzymes, such as penicillin binding proteins.

Previous work has also isolated *Pseudomonas* species with antimicrobial properties. Schlusselhuber et al. (2021) describes the isolation of a *Pseudomonas crudilactus* from raw milk that produces lipopeptides with activity against *Listeria monocytogenes* , *Staphylococcus aureus* and *Salmonella enterica.* While lipopeptides could show activity against *P. putida* and *B. subtillis (* de Souza Freitas et al., 2020; Li et al., 2020), Anti-smash analysis did not identity lipopeptides from *P. crudilactis* MMA91. The work presented here confirms antibacterial clearing by this soil isolate and suggests this bacterium has many mechanisms by which it can inhibit the growth of other bacteria. Future analysis is required to determine which of these secondary metabolites inhibit growth of *P. putida* and *B. subtilis.*

## Methods

Bacterial Isolation

*Pseudomonas crudilactis* was isolated from a soil sample in Lake Johnson State Park in Raleigh, NC (35.76241, -78.712824) as part of a microbiology research course utilizing the Tiny Earth protocols (Hernandez et al. 2022). To isolate the bacteria, one gram of soil was diluted in a sodium buffer saline and plated on Luria Broth (LB) plates using serial dilution methods.

Screening against ESKAPE organisms

Safe ESKAPE organisms: *Staphylococcus epidermidis* , *Pseudomonas putida* , *Enterobacter aerogenes* , *Mycobacterium smegmatis* , *Bacillus subtilis* , *Acinetobacter baylyi* , *Erwinia carotovora* , or *Escherichia coli,* were inoculated onto LB agar (100 uL) and then a colony of MMA91 was patched onto each safe ESKAPE lawn using the spread patch method (Hernandez et al. 2022). After 24-48 hours in 30℃, with exception for *S. epidermidis* which is grown at 37℃, plates were checked for antimicrobial activity represented by clearing.

Genomic Sequencing

DNA was extracted from MMA91 after growth in Luria Broth for 48 hours at 30℃ using the Qiagen DNAeasy UltraClean microbial kit. The genomic DNA library was performed according to the Native Barcoding Kit 24 V14 (SQK-NBD114.24) (Kolomogorov et al. 2019; https://nanopore4edu.org). The prepared library was sequencing using an Oxford Nanopore Technologies MinION instrument using a flow cell (FLO-MIN114). The draft genome assembly was performed using BV-BRC by flye version 2.9.1-b1780 and polished with racon resulting in a single contig 6,700,725 bp in length (Olson et al. 2023). Genome annotation was performed by BV-BRC using RASTtk resulting in 6,039 protein encoding sequences, 67 tRNAs, and 22 rRNAs (Chauhan and Jindal 2020). Biosynthetic gene clusters were evaluated using antiSMASH 8.0 bacterial version (Blin et al. 2025).

Genbank accession number PRJNA1466251 .

## References

[R1] Ahmed Sirwan Khalid, Hussein Safin, Qurbani Karzan, Ibrahim Radhwan Hussein, Fareeq Abdulmalik, Mahmood Kochr Ali, Mohamed Mona Gamal (2024). Antimicrobial resistance: Impacts, challenges, and future prospects. Journal of Medicine, Surgery, and Public Health.

[R2] Blin Kai, Shaw Simon, Vader Lisa, Szenei Judit, Reitz Zachary L, Augustijn Hannah E, Cediel-Becerra José D D, de Crécy-Lagard Valérie, Koetsier Robert A, Williams Sam E, Cruz-Morales Pablo, Wongwas Sopida, Segurado Luchsinger Alejandro E, Biermann Friederike, Korenskaia Aleksandra, Zdouc Mitja M, Meijer David, Terlouw Barbara R, van der Hooft Justin J J, Ziemert Nadine, Helfrich Eric J N, Masschelein Joleen, Corre Christophe, Chevrette Marc G, van Wezel Gilles P, Medema Marnix H, Weber Tilmann (2025). antiSMASH 8.0: extended gene cluster detection capabilities and analyses of chemistry, enzymology, and regulation. Nucleic Acids Research.

[R3] Cao Li, Do Truc, Link A James (2021). Mechanisms of action of ribosomally synthesized and posttranslationally modified peptides (RiPPs). Journal of Industrial Microbiology and Biotechnology.

[R4] Chandra Niharika, Kumar Sunil (2017). Antibiotics Producing Soil Microorganisms. Soil Biology.

[R5] de Souza Freitas F, Coelho de Assis Lage T, Ayupe BAL, de Paula Siqueira T, de Barros M, Tótola MR (2020). Bacillus subtilis TR47II as a source of bioactive lipopeptides against Gram-negative pathogens causing nosocomial infections.. 3 Biotech.

[R6] Hernandez, S., Tsang, T., Bascom-Slack, C., Broderick, N., & Handelsman, J. (2020). *Tiny Earth: A research guide to studentsourcing antibiotic discovery* . **ISBN ** 9798385144198.

[R7] Huang Wenqian, Wang Yingxia, Tian Weisheng, Cui Xiaoxue, Tu Pengfei, Li Jun, Shi Shepo, Liu Xiao (2022). Biosynthesis Investigations of Terpenoid, Alkaloid, and Flavonoid Antimicrobial Agents Derived from Medicinal Plants. Antibiotics.

[R8] Li Meng, Mou Haijin, Kong Qing, Zhang Tan, Fu Xiaodan (2020). Bacteriostatic effect of lipopeptides from Bacillus subtilis N-2 on Pseudomonas putida using soybean meal by solid-state fermentation. Marine Life Science & Technology.

[R9] Miller Sarah, Hernandez Paul R., Du Wenyi, Aldana Cristian Cervantes, Lee Hyewon, Maldonado Natalia, Sandoval Perla, Vong Janice, Young Gerald, Handelsman Jo, Broderick Nichole A., Estrada Mica (2025). Tiny Earth CURE Demonstrates Equitable Benefits for U.S. College Science Students. CBE—Life Sciences Education.

[R10] Prazdnova Evgeniya V., Kulikov Maxim P., Khmelevtsova Ludmila E. (2026). The Potential of Non-Ribosomal Peptide Engineering for Creating New Antimicrobial Complexes. Molecules.

[R11] Precord Timothy W., Mahanta Nilkamal, Mitchell Douglas A. (2019). Reconstitution and Substrate Specificity of the Thioether-Forming Radical
*S*
-Adenosylmethionine Enzyme in Freyrasin Biosynthesis. ACS Chemical Biology.

[R12] Schlusselhuber M, Girard L, Cousin FJ, Lood C, De Mot R, Goux D, Desmasures N (2021). Pseudomonas crudilactis sp. nov., isolated from raw milk in France.. Antonie Van Leeuwenhoek.

[R13] Wackett Lawrence P. (2016). Microbial β‐lactone natural products. Microbial Biotechnology.

[R14] Wahnou Hicham, El Kebbaj Riad, Demoré Béatrice, Limami Youness, Duval Raphaël Emmanuel (2026). Current State of the Fight Against Antimicrobial Resistance: What Are the Different Strategies for Tomorrow?. Antibiotics.

